# Knowledge, Attitude and Practice for Pruritus Management in Physicians and Patients with Diabetes

**DOI:** 10.3390/clinpract12010004

**Published:** 2022-01-05

**Authors:** Sanjay Kalra, Asit Mittal, Roheet M. Rathod, Colette Pinto, Rahul Rathod, Amey Mane

**Affiliations:** 1 Department of Endocrinology, Bharti Hospital, Karnal 132001, India; brideknl@gmail.com; 2 Department of Dermatology, R.N.T Medical College and Attached Hospitals, Udaipur 313001, India; asitmittal62@gmail.com; 3 Medical Affairs, Dr. Reddy’s Laboratories Pvt Ltd., Ameerpet, Hyderabad 500016, India; colettestephenp@drreddys.com (C.P.); rahul.rathod@drreddys.com (R.R.); amey.mane@drreddys.com (A.M.)

**Keywords:** qualitative research, knowledge, attitudes, practice, treatment, India

## Abstract

Pruritus is a common dermatological condition observed in patients with diabetes, making it a dermatometabolic condition. Being multiaethiological, pruritis is caused by autoimmune, genetic, infectious and various systemic diseases. The present survey aimed to understand the knowledge, attitude and practice toward pruritus among Indian physicians and patients with diabetes presenting with pruritus. A telephonic, cross-sectional, qualitative survey was conducted among physicians and patients across five cities in India from July–August 2020. An open-ended discussion guide was used for the interview; the data were analyzed to check for common themes and trends. A majority of the consulting physicians (CPs) believed that uncontrolled diabetes is the main causal factor for pruritus in patients with diabetes and reported that currently there are no standard tests or treatment guidelines for its management. CPs emphasized proper monitoring and counseling to overcome current challenges. Patients reported a negative impact of pruritus on their daily activities and quality of life. The survey concluded that poor management of diabetes is one of the main causal factors for patients with diabetes presenting with pruritus in India. CPs emphasized controlling diabetes along with symptomatic treatment. For patients, pruritus has multifaceted effects on their health, overall well-being, and quality of life.

## 1. Introduction

Pruritus or itching is a common dermatological condition characterized by an unpleasant sensation of the skin that provokes the urge to scratch [1]. It is caused by a variety of dermatological diseases ranging from autoimmune, genetic, and infectious to various systemic diseases such as endocrine and metabolic disorders, neoplastic and hematological disorders, pregnancy, and a few medications [2]. An estimated 3–50% patients with diabetes may present with generalized pruritus [3].

Global evidence suggests that the prevalence of pruritus associated with diabetes ranges between 18.4–27.5% [3,4,5,6]. An Indian observational study reported pruritus as a primary symptom in 50% patients with diabetes [7], while another Indian cross-sectional study conducted in a tertiary care hospital reported pruritus in 13.3% of the patients with diabetes without skin lesions [8].

Underlying pathophysiology, comorbidities and medications predispose patients with diabetes to develop pruritus [5]. Diabetes is associated with impaired skin elasticity and decreased cutaneous sebaceous gland activity [9]. Several studies have linked increased blood glucose levels with development and severity of pruritus [3,5,10]. Pruritus associated with diabetes can be considered a dermatometabolic condition and is often localized rather than generalized [4,6,11,12]. Different evaluation techniques are used to determine pruritis in different conditions. Psoriasis is determined with the Epworth Sleepiness Scale, Itch- visual analogue scheme, the Dermatology Quality of Life Index, and Psoriasis Area Severity Index. The main comorbidities in pruritis including diabetes are synthesized using the Charlson comorbidity index [13,14]. Additionally, pruritis was determined to be the most frequent dermatological condition in lung cancer and melanoma patients using immune checkpoint inhibitors ranging from grade 1–4 depending on the therapy used [15,16].

The current management approach for pruritus includes therapies that provide symptomatic relief. Therapy for chronic generalized pruritus includes various topical agents such as anesthetics, antipruritics, emollients, cooling agents, and corticosteroids. Further, systemic treatments for symptomatic relief include antihistamines, an opioid receptor antagonist, corticosteroids and phototherapy [17,18]. For the management of pruritus associated with diabetes, better control of glucose levels may be considered instrumental [3].

Presently, there are no global and Indian standard treatment protocols or guidelines for treating diabetes patients presenting with pruritus [19,20]. This may lead to inconsistent management approaches by physicians. Additionally, an existing high pill burden in patients with diabetes and added comorbidities may lead to lack of adherence to treatment [21]. From a patient’s perspective, a simple itch can have lasting psychological and social effects [22], and can affect patients’ quality of life [23]. Patients with pruritus are often unsure of the seriousness of their condition and are unable to decide when to seek medical attention [24].

Although, pruritus is a common manifestation of diabetes, there is a lack of global and India-specific evidence to understand the perception of physicians and patients with diabetes toward pruritus. Hence, to assess the knowledge, attitudes, and practice among physicians and patients with diabetes mellitus regarding pruritus, this qualitative survey was conducted in India.

## 2. Materials and Methods

### 2.1. Study Design

A telephonic, cross-sectional, qualitative survey was conducted among consulting physicians (CPs) and patients with diabetes from July–August 2020 across five cities (Delhi, Mumbai, Bangalore, Hyderabad, and Chennai) in India. The CPs were identified using the IQVIA OneKey^®^ Healthcare Industry database and the selected CPs suggested the names of the patients that were contacted for the survey. The inclusion criteria for the CPs were-Doctor of Medicine (MD) physician treating diabetes, practicing for more than 15 years and treating more than 100 patients per month. The inclusion criteria for the patients were-male and female patients aged between 35–65 years with diabetes for more than 5 years and having pruritus or itch for the past 1 year. A series of screening questions were asked to select the CPs and patients, fulfilling the inclusion criteria. A total of 10 s CP and 10 patients were selected for the survey (2 per city).

The survey was approved by the Conscience Independent Ethics Committee, Ahmedabad, Gujrat, India. Verbal consent was obtained from the CPs and patients prior to conducting the interview. The participants (CPs and patients) were fairly compensated for their time in accordance with the Market Research Society of India [25] and IQVIA standard practices.

### 2.2. Methodology

The telephonic interviews were conducted by representatives of the clinical research organization (IQVIA) using a discussion guide with open-ended questions in English language. The overall duration of the interview was 40–45 min. The CPs were interviewed to understand patient dynamics, existing knowledge, attitude toward management, and management practices related to pruritus. The patients were interviewed to explore their understanding of symptoms, diagnosis and treatment options, attitude toward management, and challenges in the management of pruritus.

### 2.3. Data Analysis/Evaluation

The interview was audio-recorded, and the responses were transcribed, segregated, and analyzed to check for common themes and trends. The analysis was based on the interviews and feedback from the participants. Patient and CPs’ perceptions around severity of pruritus associated with diabetes as compared to fever, weakness, cough, and cold was charted on a scale of 1 to 5 where 1 = not at all severe and 5 = extremely severe. Patient and CP satisfaction with current available treatment was also collected using a similar scale, where 1 = not at all satisfied and 5 = extremely satisfied.

All the identified trends were then categorized separately for the CPs and patients. The main identified themes were divided on the basis of knowledge, attitude, and practice, i.e., management of pruritus by CP and patient.

## 3. Results

### 3.1. Consulting Physician Practice Dynamics

The CPs (*n* = 10) treated approximately 570 patients in a month irrespective of the indication, out of which 43% (*n* = 274) were patients with diabetes. Of the patients with diabetes, 38% (*n* = 95) were suffering from dermatological conditions and 55% (*n* = 50) of those patients had pruritus. The CPs stated that they treated a majority of the patients (70%) with pruritis who consulted them and referred only 30% (commonly to a dermatologist).

### 3.2. Knowledge around Pruritus Associated with Diabetes

#### 3.2.1. Correlation between Pruritus and Diabetes

All the CPs responded that they had seen pruritus in their patients with type 2 diabetes. A majority of the CPs believed that uncontrolled diabetes leads to inflammatory changes in the body, resulting in dermatological issues such as secondary infections, pruritus, rashes, etc. The CPs put forth three possible etiologies for pruritus and diabetes: (1) long-standing diabetes results in drying of skin, which can further precipitate itching and scratching, especially during winters, (2) Micro-angiopathy, i.e., peripheral blood vessels not supplying the proper amount of blood to skin tissues and this decrease in blood supply resulting in itching and (3) infections due to an excessive amount of blood glucose acting as a culture medium for yeast or other related infectious organisms.

#### 3.2.2. Types of Pruritus

CPs responded that based on the severity and duration of pruritus, chronic pruritus forms the major type of pruritus (60%), with symptoms lasting for more than 3 weeks.

#### 3.2.3. Decision on Treatment and Diagnostic Tests

All the CPs agreed that there is no standard treatment guideline for the management of pruritus in patients with diabetes in India. The CPs devised the treatment protocols for patients based on the type of pruritus-localized or generalized. Further, since the diagnosis was based on their expertise and patient history, and in the absence of a specific test, tests were done to determine the underlying cause. The CPs preferred diagnostic tests for generalized pruritus. The common diagnostic tests included complete blood count, liver function, renal function test, congenital pruritus C1 esterase deficiency, serum immunoglobulin, antinuclear antibody (ANA), thyroid test, C-reactive protein test and immunoglobulin E (Ig E) levels. In the case of acute symptoms, CPs sometimes preferred to recommend ANA tests along with immunoglobulin E (IgE) to make sure of the presence or absence of allergies.

#### 3.2.4. Profile of Patients with Pruritus

Most of the patients were aged around 40 years and had diabetes for the past 4–10 years. The usual sites for pruritus were genitals, abdomen, and buttocks. Pruritus was more common in female obese patients who were working and leading a very stressful lifestyle and in patients with an uncontrolled glycemic level with HbA1c of >9. Pruritus was rarely observed in patients with type 1 diabetes mellitus. The typical patient profile did not vary in acute and chronic pruritus.

### 3.3. Attitude toward Management of Pruritus

#### 3.3.1. Opinion on Pruritus

The CPs stated that patients resistant to insulin and suffering from chronic pruritus were challenging to manage. A majority (60%) of CPs agreed that bringing diabetes under control also resulted in controlling pruritus, 30% were unsure of that, and only 10% disagreed about the diabetes control for pruritus management.

#### 3.3.2. Perceptions around Severity of Pruritus Associated with Diabetes

The CPs rated their perception toward severity of pruritus as 3.5/5. The reasons for this perception included difficulty in identifying the underlying cause of pruritus, physical discomfort caused to patients, and hindrance in carrying out day-to-day activities for patients.

#### 3.3.3. Patient Education and Counseling

CPs gave lower priority to information about pruritus during diagnosis of diabetes because they believed that there were other aspects of diabetes that were much more crucial to be discussed. Further, not all diabetes patients develop pruritus, and hence counseling for pruritus was reserved for when patients presented with symptoms. All the CPs stated that counseling was required for pruritus; however, the stage at which it was to be included varied. Few CPs claimed that discussion on pruritus during initial counseling helped with patients taking measures to prevent it.

### 3.4. Practice in Management of Pruritus

#### 3.4.1. Proportion of Patients Recommended Treatment

The CPs revealed that they recommended treatment to 60–70% of the patients, out of which 80% patients were compliant about the treatment. The treatment recommendation depended on the intensity of symptoms. Only 5–10% of the patients were recommended proactive treatment, whereas a majority were advised with reactive treatment. The main treatment goals were symptomatic relief for patients and treating the causative factor. Various factors considered by the CPs while choosing treatment regime for patients included blood glucose levels, age, socioeconomic condition of patients, severity of pruritus, site of pruritus, existing infection, and allergies, and other comorbidities were also considered important. The CPs rated satisfaction with current treatment as 4/5 (Table 1).

#### 3.4.2. Challenges Encountered with Current Management of Pruritus

The CPs said that patients were reluctant to take long-term treatment because it added to their cost of therapy and pill burden. Overall, unavailability of medication options for severe and chronic pruritus patients led to the use of steroids. Expensive diagnostic tests and difficulty in clinical examinations formed another challenge, especially among patients with a delayed diagnosis. Sedation due to antihistamines has often been reported by patients, and there can be recurrence on discontinuation of treatment and aggravated symptoms due to seasonal changes, especially during winters (Table 2).

#### 3.4.3. Ways to Overcome the Current Challenges

CPs emphasized proper monitoring and regular follow-up with patients along with counseling sessions and identifying mistakes that might lead to recurrence. They also added that it is essential to ensure that there are no drug–drug or food–drug interactions that may further deteriorate the patient’s condition. CPs added that increasing awareness leading to early diagnosis, educating physicians treating diabetes patients, i.e., family physicians and general physicians, to better understand the correlation between diabetes and pruritus can be beneficial.

#### 3.4.4. Participation in Continuous Medical Education (CME’s) by Physicians

A total of 40% CPs attended CMEs on pruritus or participated as speakers. Overall, dermatologists had higher involvement with pruritus CMEs compared to diabetologists and CPs.

#### 3.4.5. Patient Baseline Characteristics

Of the total patients (*n* = 10), 60% were males and 40% were females. The males were predominantly in the age group of 45–65 years, and females were in the age group of 40–55 years. A majority of the males were diagnosed with diabetes over the past 5–10 years, and females had been suffering from diabetes for 5–8 years. Male patients had reported itching in the past 1–1.5 years. Apart from regular doctor consultations and medications, female participants tried to maintain their glucose levels with lifestyle changes. Other comorbidities seen in the patients were hypertension, hypercholesteremia, obesity, and skin problems such as itching, dryness, burning feet, and fordyce.

### 3.5. Knowledge around Pruritus Associated with Diabetes

#### Patients’ Initial View toward Pruritus

Majority of the patients did not consider itching as a serious issue and tried alternative therapies including homeopathic medicines, ayurvedic medicines and home remedies for a couple of weeks before approaching a doctor. The trigger to seek medical attention was the progression of itching and visible redness. A majority of patients visited a treating diabetologist/CP, and a few visited a general physician/family physician and a dermatologist.

### 3.6. Attitude toward Management of Pruritus

#### 3.6.1. Patients’ Journey

The patients reported symptoms including dryness, rashes, and itching around arms, legs, feet, armpits, and the abdomen, which they dealt with for 1–2 months before approaching the doctor. The patients further added that on reporting symptoms, their physicians counseled them on pruritus and its relationship with diabetes and lifestyle practices. A majority of the patients claimed that they did not face any side effects with any medication that was prescribed for pruritus; however, they fear ill effects on their kidney or liver with long-term usage. For minor side effects, such as drowsiness, patients consulted the doctor and got the dose adjusted.

Patients continued using lotions and creams prescribed by their physicians as that provided relief and did not hinder daily functioning, while they did consult their physicians if the symptoms elevated. They found it bothersome and embarrassing when pruritus was seen on their face, scalp or exposed part of the body, and after short relief they discontinued their medication due to pill burden (Table 3).

#### 3.6.2. Perception on Severity of Pruritus

The patients rated their perception toward severity of pruritus as 4, i.e., a more severe condition when comparing it with fever, weakness, cough, and cold. The reasons for this perception included the hindrance pruritus caused in their day-to-day activities.

### 3.7. Practice of Management of Pruritus

#### 3.7.1. Current Treatment Options

The satisfaction level with current treatment options was rated at 4.3 by the patients. The challenges encountered by patients were multifaceted, including the psychological impact of visible symptoms, social embarrassment due to itching, and hindrance in daily activities. Patients also had restricted mobility owing to itching on friction/abrasions and had to change clothes more frequently. Some patients also reported skin shedding and bleeding after itching, which led to them feeling uncomfortable to the extent of leading to absenteeism at work. On their physician’s advice they avoided wearing tight clothes, applied creams and lotions prescribed to keep skin moisturized, and increased their fluid intake to keep their bodies cool.

#### 3.7.2. Patient’s View on Patient Support Program

Only a small proportion of patients had enrolled themselves for patients support programs, while a majority (80%) had not enrolled as they were satisfied with their current treatment. The patient support programs were mostly for diabetes, but in cases of associated conditions they additionally assisted patients with better monitoring of their progress.

## 4. Discussion

Diabetes is a metabolic disorder, and its underlying pathophysiology along with comorbidities predispose patients to develop pruritus [5]. Previously published literature has established that pruritus is a common manifestation of diabetes [3,17]. Furthermore, pruritis even affects the sleep pattern and circadian rhythm in patients with diabetes. Patients experience more itch in N1, N2, and REM sleep. Studies have suggested lack of sleep to be associated with metabolic dysregulations including diabetes [26]. It was also observed that in cases where disruption in circadian rhythm occurs, there is a higher chance of developing conditions such as psoriasis [27]. Dermatological conditions including pruritis also lead to significant loss of compliance [28].

In the present survey, all the CPs reported having seen pruritus in their type 2 diabetes patients.

Further, CPs reported that patients with unmanaged diabetes have a predisposition toward developing pruritus that can be linked to the inflammatory changes caused by high glucose levels. The finding was in line with earlier published studies that also attributed elevated blood glucose levels with the development of pruritus [3,5,10], thus highlighting the key causes and steps for the management of pruritus in patients with diabetes-reduction in elevated blood glucose levels along with changes in lifestyle.

Pruritus can have varied etiology, and with the lack of any specific diagnostic tests physicians often find it difficult to diagnose [19,20]. CPs reported giving importance to their own expertise and patient history to ascertain the etiology and suggest suitable treatment to the patient.

Pruritus can present as localized or generalized, and as an acute or chronic condition [17]. Often, treatment is recommended for chronic pruritus; however, the treatment choice has a multifaceted approach, with importance given to providing symptomatic relief to the patient and also treating the underlying cause for pruritus [17,19]. The physicians in the present survey suggested the use of various skin-directed and systemic treatment options for symptomatic relief, highlighting management of uncontrolled diabetes as being the primary goal for long-term management of pruritus.

The current survey indicated that Indian physicians did not give high priority to a discussion about pruritus during the initial diagnosis of diabetes, as all patients may not develop it. Moreover, with limited time physicians preferred to discuss managing diabetes and lifestyle changes at the time of initial diagnosis.

The key challenges in managing patients with pruritus due to diabetes by CPs in the present survey include the existing pill burden on patients with diabetes and various other comorbidities, which are already established challenges for the patient treatment adherence [21]. Additionally, with the cost of therapy associated with already prescribed medications being a burden for patients [29], treatment for pruritus aggravates the situation. Due to lack of current treatment guidelines in place for management of pruritus presenting in diabetes patients, physicians do not have a standardized regimen in place. Other challenges highlighted by the CPs include expensive tests and difficulty in clinical examinations, especially among patients delaying diagnosis for a longer time.

From the patients’ perspective, they approached doctors after progression of itching or due to visible redness. This reflects the lack of awareness about the condition among Indian patients with diabetes. Further, due to a delay in seeking medical care the diagnosis and treatment are adversely affected.

The effective management of pruritus involves multiple stakeholders, and steps need to be taken by physicians as well as the patients. The physicians can work on having regular follow-ups with patients, proper monitoring, and counseling sessions with patients; further, they must keep themselves updated by actively participating in conferences and CME initiatives. The patients with the right awareness must seek medical attention early at the onset of the symptoms so as to facilitate early diagnosis and treatment.

The current survey used a small heterogeneous sample, so the results may not generalize to the entire population in India and should be used for directional purposes only. In addition, due to a smaller sample size, results were not validated for inter individual variations. However, in-depth probing questions were asked to obtain maximum details regarding the research questions, and detailed information was obtained regarding the reasons for a certain opinion of CPs and patients. Despite the aforesaid limitation, this was the first of its kind survey providing valuable insights about the CPs’ and patients’ perspectives toward pruritus associated with diabetes and established the need to address the gap in treating pruritus in diabetes patients in India. The results from this survey can form the base of further large-scale studies in India.

## 5. Conclusions

The survey concluded that Indian physicians perceived poor management of diabetes as a primary cause of pruritus along with lack of proper hygiene and stress. The authors concluded that Indian physicians perceived poor management of diabetes as a primary cause of pruritus. As the mechanism, the changes in receptors for cutaneous peripheral nerve free endings have been reported in addition to the dry skin, micro-angiopathy, and infections in recent research. Indian physicians advise symptomatic treatment to the patients; however, it has been established that treating the cause is more important. From the patients’ perspective pruritus is a severe condition; however, they did not visit doctors at the initial stage and tried to alleviate symptoms using alternate therapies. The patients are interested in managing symptoms so that the condition does not hinder their day-to-day life. There is need for educating and motivating the patients to take counseling so as to better manage their condition.

## Figures and Tables

**Table 1:** Treatment prescribed, duration, and dosage of treatment.

Treatment initiation	
• Antihistamines ✓ First Generation: Cetirizine ✓ Second Generation: Desloratadine, Fexofenadine, Olopatadine, Loratadine • Ointments	
Severe pruritus	
Gabapentin, Pregabalin, Prothiaden, or Duloxetine are used in combination with another topical agent Antibiotics such as Zanocin, Itromed Steroids like Prednisolone if patients are not responding to antihistamines	
Duration and dosage	
Acute pruritus	Treatment for 1 week to 10 days Dosage depends on severity of pruritus Given for 1–2 times a day
Chronic pruritus	Treatment done for 2–3 months Patients’ tolerability to medicines is monitored

**Table 2:** Select physicians’ responses.

Domain	Theme	Participants	Responses
Knowledge	Diabetes and associated conditions	Consulting physician, Delhi	“I mean diabetes, has got lot of skin manifestations. It could be both infective as well as non-infective. And since diabetes is one of the very important areas of my practice, so I see lot of infective patients of diabetes which are primarily you know Candida infections, or it could be pyodermas. And then there are non-infectious cause like your very commonly cause is, diabetic dermopathy is the word that is given. And then something which is very special for diabetes, not commonly seen is necrobiosis and then you find rare conditions like granuloma annulare. And I think the most common condition which I did not highlight, or post is a patient coming with simple itching and no other manifestation. You may find that the patient has got very dry skin, which is called as xerosis. And I feel xerosis is the most common manifestations of diabetic as far as the skin involvement is concerned.”
	Pruritus relation with diabetes	Consulting physician, Mumbai	“Pruritus and diabetes, in diabetes the people are prone get infections, and in that case, they may have pruritus. But it is not hard and fast rule that each and every person who is having diabetes must have pruritus.”
	Types of pruritus	Consulting physician, Bangalore	“Commonest is localized pruritus, either it could be in the toes or in the skin folds.”
	Treatment Guidelines	Consulting physician, Delhi	“Whatever I have read in my books, I try to practice that. I am not aware of any recent guidelines really speaking.”
Attitude	Perception about severity		“I don’t think there is a direct relation, to prevent further complications we have to keep a track on the diabetes patient and see if they are developing any complication. Only regular monitoring and tracking can help in such patients.”
Practice	Treatments Prescribed	Consulting physician, Mumbai	“Chronic patients are normally about elderly, so I would start with prednisolone 30 mg, for the first 5 days and then start tapering it off quickly. So, that they do not have any other abnormality, like glycemic control going haywire, the blood pressure going haywire and they end up developing symptoms like muscle weakness, increased appetite so all those things.”
		Consulting physician, Bangalore	“If you have to treat them for chronic pruritus, we have to give them steroid. When steroid is given, blood glucose goes high & managing blood glucose gets difficult.”
	Satisfaction with current treatment	Consulting physician, Bangalore	“If you have to treat them for chronic especially, we have to give them steroid. When you give steroid, their blood glucose goes high, managing that in the presence of the steroids becomes difficult. Secondly, the long-term management that requires, say 2 weeks, 4 weeks and all that. The patients may not be willing to take. The discontinuation in treatment that happens.”

**Table 3:** Medications taken by patients.

Class of Medication	Medication
Oral Medications	Antihistamines: Cetirizine, Avil, Allegra (Avil was frequently associated with side effects) Antibiotics such as Itromed and Zanocin are used to control the infections Uprise D3 for Vitamin D and Calcium deficiency
Topical Applications (Emollient, Steroids, Moisturizers, Creams and lotions, Powders)	Calcitriol, Venusia, Lactocalmine Lotions Moiztal, Tanglow, Emoderm creams Erytop oil Benadryl ointment Telovet ointment DermaDay Soap Clotrimazole and Candida Powders

## Data Availability

No new data were created or analyzed in this study. Data sharing is not applicable to this article.
